# Self-regulation strategy, feedback timing and hemodynamic properties modulate learning in a simulated fMRI neurofeedback environment

**DOI:** 10.1371/journal.pcbi.1005681

**Published:** 2017-07-28

**Authors:** Ethan F. Oblak, Jarrod A. Lewis-Peacock, James S. Sulzer

**Affiliations:** 1 Department of Mechanical Engineering, The University of Texas at Austin, Austin, Texas, USA; 2 Department of Psychology, The University of Texas at Austin, Austin, Texas, USA; 3 Institute for Neuroscience, The University of Texas at Austin, Austin, Texas, USA; Harvard University, UNITED STATES

## Abstract

Direct manipulation of brain activity can be used to investigate causal brain-behavior relationships. Current noninvasive neural stimulation techniques are too coarse to manipulate behaviors that correlate with fine-grained spatial patterns recorded by fMRI. However, these activity patterns can be manipulated by having people learn to self-regulate their own recorded neural activity. This technique, known as fMRI neurofeedback, faces challenges as many participants are unable to self-regulate. The causes of this non-responder effect are not well understood due to the cost and complexity of such investigation in the MRI scanner. Here, we investigated the temporal dynamics of the hemodynamic response measured by fMRI as a potential cause of the non-responder effect. Learning to self-regulate the hemodynamic response involves a difficult temporal credit-assignment problem because this signal is both delayed and blurred over time. Two factors critical to this problem are the prescribed self-regulation strategy (cognitive or automatic) and feedback timing (continuous or intermittent). Here, we sought to evaluate how these factors interact with the temporal dynamics of fMRI without using the MRI scanner. We first examined the role of cognitive strategies by having participants learn to regulate a simulated neurofeedback signal using a unidimensional strategy: pressing one of two buttons to rotate a visual grating that stimulates a model of visual cortex. Under these conditions, continuous feedback led to faster regulation compared to intermittent feedback. Yet, since many neurofeedback studies prescribe implicit self-regulation strategies, we created a computational model of automatic reward-based learning to examine whether this result held true for automatic processing. When feedback was delayed and blurred based on the hemodynamics of fMRI, this model learned more reliably from intermittent feedback compared to continuous feedback. These results suggest that different self-regulation mechanisms prefer different feedback timings, and that these factors can be effectively explored and optimized via simulation prior to deployment in the MRI scanner.

## Author summary

By repeatedly stimulating fine-grained patterns of neural activity, it is possible to manipulate behaviors associated with these patterns. While millimeter-scale patterns cannot yet be targeted with noninvasive brain stimulation, some people can learn to self-stimulate these activity patterns if they receive real-time feedback of their own recorded brain activity through a procedure known as fMRI neurofeedback. Other ‘non-responders’ are, for reasons unknown, unable to learn how to self-regulate these patterns. Here, we investigate how the properties of the fMRI signal, feedback timing, and self-regulation strategies may lead to this non-responder effect. The signal recorded by fMRI is related to blood flow in the brain and can be delayed by up to six seconds relative to underlying neural activity, which makes it difficult to learn. Because experiments in the MRI scanner are costly and time-consuming, we created a simulated neurofeedback environment to compare continuous versus intermittent feedback timing and cognitive versus automatic self-regulation strategies. In a cognitive experiment with human participants playing a simple game with the simulated neurofeedback signal, we found continuous feedback led to faster learning. However, in a computer model of automatic reward-based learning, we found that intermittent feedback was more reliable. These results will help improve future fMRI neurofeedback experiments and treatments by improving the efficacy of neurofeedback training procedures.

## Introduction

Neuroimaging studies often find correlations between behaviors and recorded neural activation. To test whether these relationships are causal, neuroscientists must find a way to manipulate the underlying neural structure and observe whether the associated behavior is affected. In human neuroscience research, we are usually limited to noninvasive methods that have minimal associated risk. Currently, safe and noninvasive neural stimulation techniques such as transcranial magnetic stimulation (TMS) and transcranial direct-current stimulation (tDCS) can only affect neural structures on the scale of centimeters [1, 2]. These techniques are too coarse to manipulate behaviors that correlate with the millimeter-scale patterns of neural activation recorded by functional magnetic resonance imaging (fMRI). Using multi-voxel pattern analysis (MVPA [3]), we can identify neural features such as orientation tuning [4, 5] and complex motor programs [6, 7] which are inaccessible to other human neuroimaging analysis methods.

**Fig 1:**
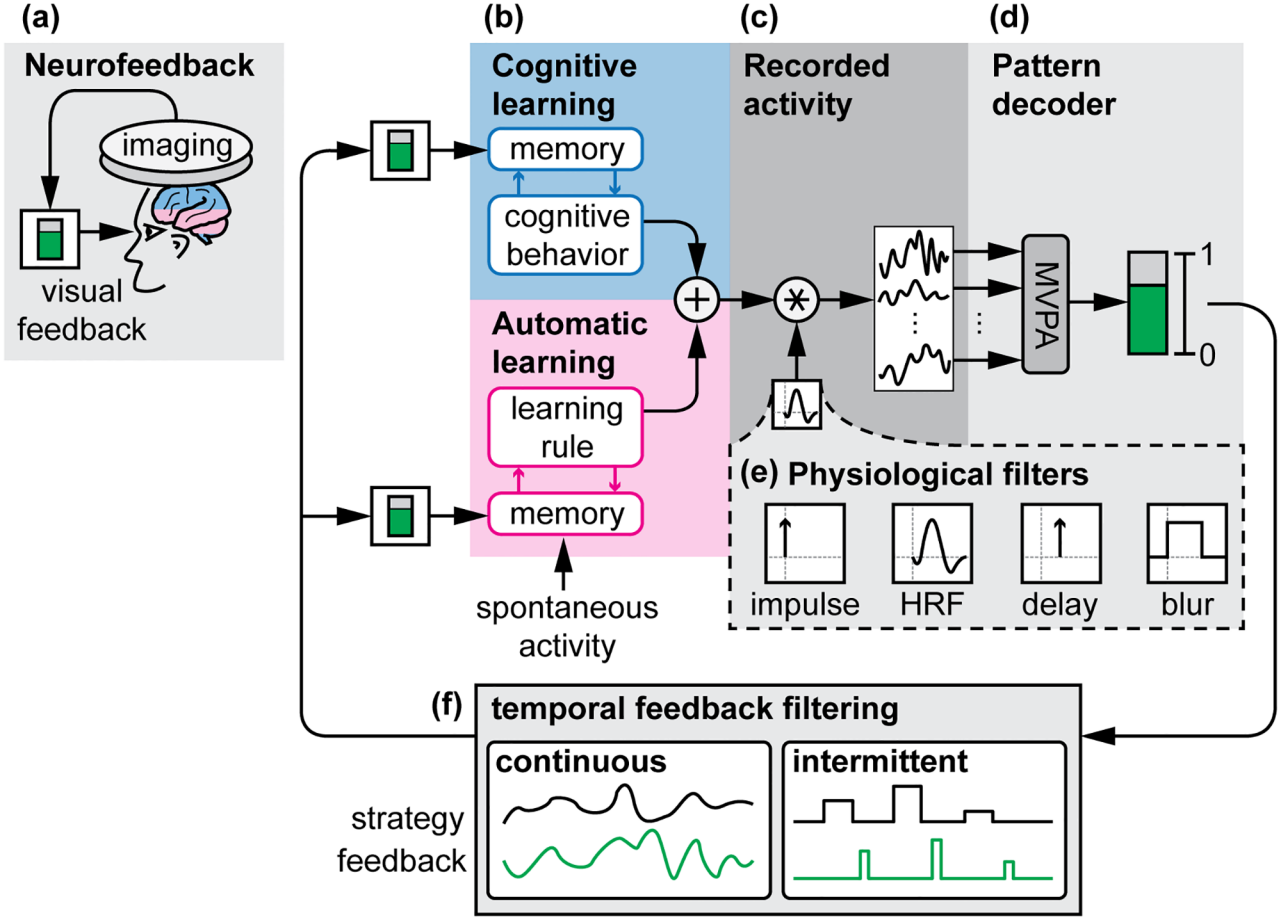
System model of neurofeedback. **(A)** Neurofeedback is biofeedback of neural activity, commonly presented as a visual thermometer (in green) that participants learn to control. **(B)** Neurofeedback is learned through either cognitive or automatic learning processes. In cognitive learning, participants try various cognitive behaviors and, through trial-and-error, must learn which behaviors result in successful regulation of the feedback signal. In automatic learning, spontaneous neural activity is held in memory, and the feedback signal reinforces this neural activity according to an internal learning rule. **(C)** Underlying neural activity is convolved with a physiological filter such as the hemodynamic response function (HRF) before being recorded by neuroimaging. **(D)** A multivariate pattern analysis (MVPA) classifier is used to convert multiple channels (or voxels) of neural activity into a single value that represents how closely the recorded neural activity matches a desired pattern. **(E)** We present four candidate physiological filters: an instantaneous impulse, the canonical HRF, a 6-second delay, and a 10-second moving average blur. **(F)** Once decoded, feedback can either be presented continuously or intermittently.

Although we cannot exogenously stimulate these patterns, it is possible for people to endogenously activate them if they are presented with visual feedback of the recorded activity pattern in a procedure known as fMRI neurofeedback [8]. In this procedure, people must learn to elicit a targeted neural activation pattern by observing a feedback signal and adjusting their strategy to maximize this signal (Fig 1A). This technique faces challenges as many participants are unable to self-regulate. This “non-responder effect” has been quantified in electroencephalography (EEG) neurofeedback, with 15–30% of neurofeedback participants being completely unable to self-regulate [9, 10]. EEG researchers have correlated this effect with resting-state activity [11], white matter tract integrity [12], technological knowledge [13], and sense of agency [14].

For fMRI neurofeedback, the causes of the non-responder effect have not been identified due to the cost and complexity of examining all possible factors in the MRI scanner. We believe that the characteristics of the hemodynamic response measured by fMRI are critical to understanding how people learn to self-regulate because the hemodynamic response is both delayed and blurred in time relative to underlying neural activation [15, 16]. This forms a difficult credit-assignment problem, in which participants must remember which previous behavior or underlying neural activity caused the delayed and blurred feedback to change in order to learn to self-regulate. When people explore different cognitive strategies in an attempt to control the neurofeedback signal, they must remember a time history of behaviors and determine which behavior was responsible for influencing the feedback signal (Fig 1B, top). To “remember” past neural activity, automatic learning circuits must hold spontaneous activity in memory [17] and reinforce it using an internal learning rule that is not cognitively accessible to the participant (Fig 1B, bottom).

The difficulty of this credit-assignment task could be reduced by appropriately scheduling feedback to account for the hemodynamic signal properties. One of the controversies in fMRI neurofeedback is whether to schedule feedback continuously or intermittently (Fig 1F), trading off feedback frequency to account for the hemodynamic response [18]. Only two small pilot studies have been published to address this controversy. The first study compared continuous to intermittent presentation of fMRI neurofeedback of regional activation levels from the supplementary motor area, finding that intermittent feedback produced better learning [19]. The second study found mixed results in auditory cortex, with continuous feedback showing better performance over multiple sessions [20]. It is difficult to generalize these results because success may depend on other factors such as neurofeedback signal quality and self-regulation strategy. An exhaustive search of parameters within the MRI scanner is impractical due to cost. Therefore, our main goal here was to bridge the gap in understanding neurofeedback learning by testing key factors (feedback timing and self-regulation strategy) in a simulation environment outside of the MRI scanner.

**Fig 2:**
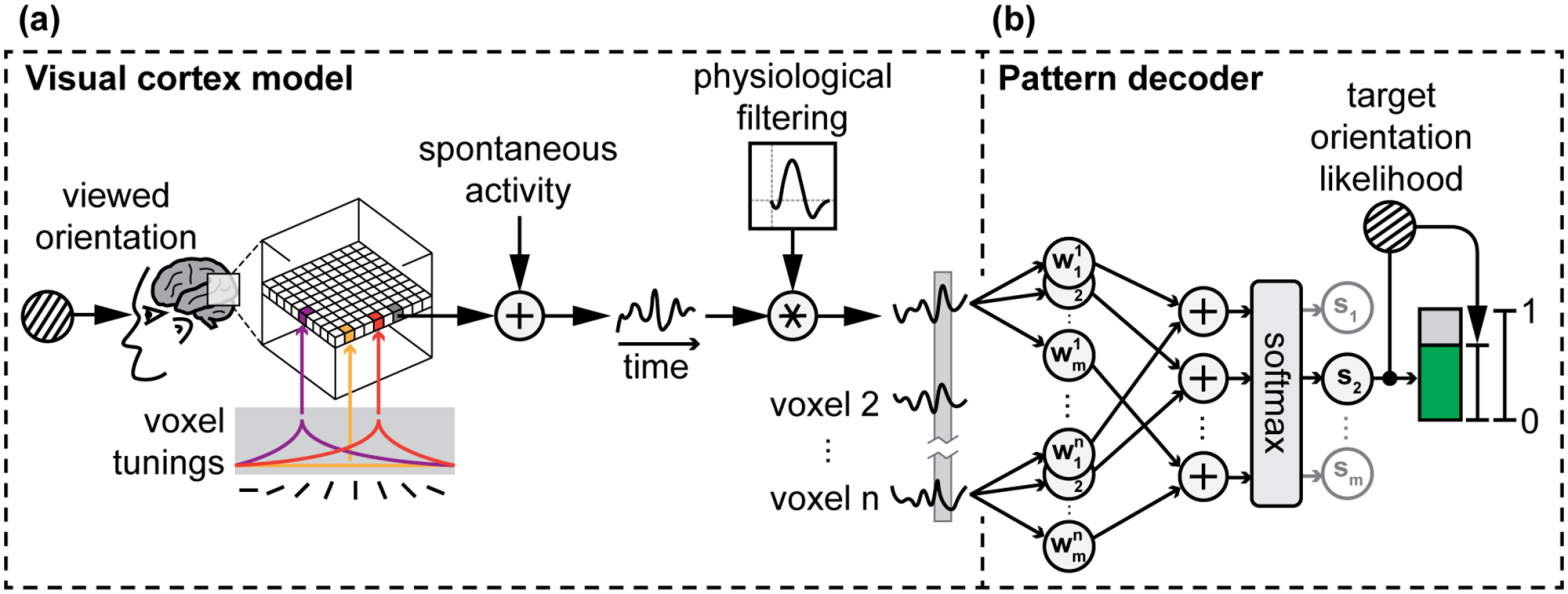
Simulated visual cortex as measured by fMRI and decoded using MVPA. **(A)** Voxels (n = 1000) in the model are either tuned to one of eight orientations (0°, 22.5°, …, 157.5°; 2.5% of voxels each), or have no orientation selectivity (80% of all voxels). All voxels have additive spontaneous activity and are convolved in time with a physiological filter such as the canonical HRF (for all candidate filters, see Fig 1e). **(B)** The pattern decoder transforms the measured pattern of simulated activity into a number indicating the likelihood that the activity matches the pattern associated with one of m target grating orientations. For cognitive experiments, m = 8 (0°, 22.5°, …, 157.5°); for automatic experiments, m = 3 (10°, 70°, 130°).

We constructed a model of early visual cortex (V1) to use as a simulated neurofeedback testbed (Fig 2A). We chose V1 as our model because this is one of the most well-studied cortical areas, starting with early electrophysiological studies in animals [21] and extending to modern fMRI studies [22]. We specifically modeled the response of V1 to grating orientations because this has been extensively studied with fMRI [4, 5, 23]. To deliver feedback from this model, we constructed similar MVPA classifiers (Fig 2B) to decode grating-associated patterns during explicit presentation of stimuli [24] as well as in the absence of stimuli [25].

To model the temporal properties of the hemodynamic response, we created four different physiological filters to apply to our simulated neural signal (Fig 1E). The first is an impulse response, which corresponds to no delaying or blurring of the recorded signal, as if we could directly record electrophysiological activity at the spatial resolution of fMRI. Next, we simulated the fMRI blood-oxygen-level-dependent (BOLD) response, using the canonical hemodynamic response function (HRF) [15] to blur and delay the underlying neural activity. The final two filters were a 6-second delay and a 10-second moving average blur, corresponding to the delay-to-peak of the HRF and a boxcar function centered around this peak, respectively. We used these two synthetic filters to investigate the unique contribution of delay and blur to the learning of fMRI neurofeedback signals. We hypothesized that isolated delay and blur responses would be more difficult to learn than an impulse response, and that the combined delay and blur of the HRF would be the most difficult.

Using our model of V1 and the four candidate physiological filters, we first examined cognitive strategies as these are the most common type of strategy given in traditional fMRI neurofeedback experiments [8, 18]. These strategies commonly take the form of mental imagery [26], which is notoriously difficult to quantify and relies on participants to self-report their strategy. To avoid this difficulty, we used a motor strategy as a proxy for mental imagery. Participants pressed one of two buttons to rotate a grating image that stimulated our V1 model. Participants had to discover a target orientation using a neurofeedback signal from our model that was filtered by one of the physiological filters. This allowed us to examine how people make cognitive decisions when faced with delayed or blurred feedback.

Next, we used the same model and filters to examine an alternative approach to fMRI neurofeedback: relying entirely on automatic learning circuits to elicit patterns of fMRI activity without requiring cognitive effort. This procedure, known as reinforcement learning or operant conditioning of fMRI patterns, has been successfully used to train the fMRI correlates of visual perception [25], confidence judgments [27], facial preference [28], color perception [29], and fear [30]. The mechanisms underlying this learning process are unclear. Because we had more factors to examine than would be feasible with the MRI scanner, we constructed a fully computational model to address how this process may occur. This allowed us to quickly and effectively evaluate whether continuous or intermittent feedback would more reliably elicit this type of automatic learning. Thus, we were able to use the same simulated neurofeedback environment to examine both cognitive and automatic learning mechanisms, demonstrating its efficacy as a neurofeedback research testbed.

## Results

### Using cognitive strategies to activate simulated visual cortex

**Fig 3:**
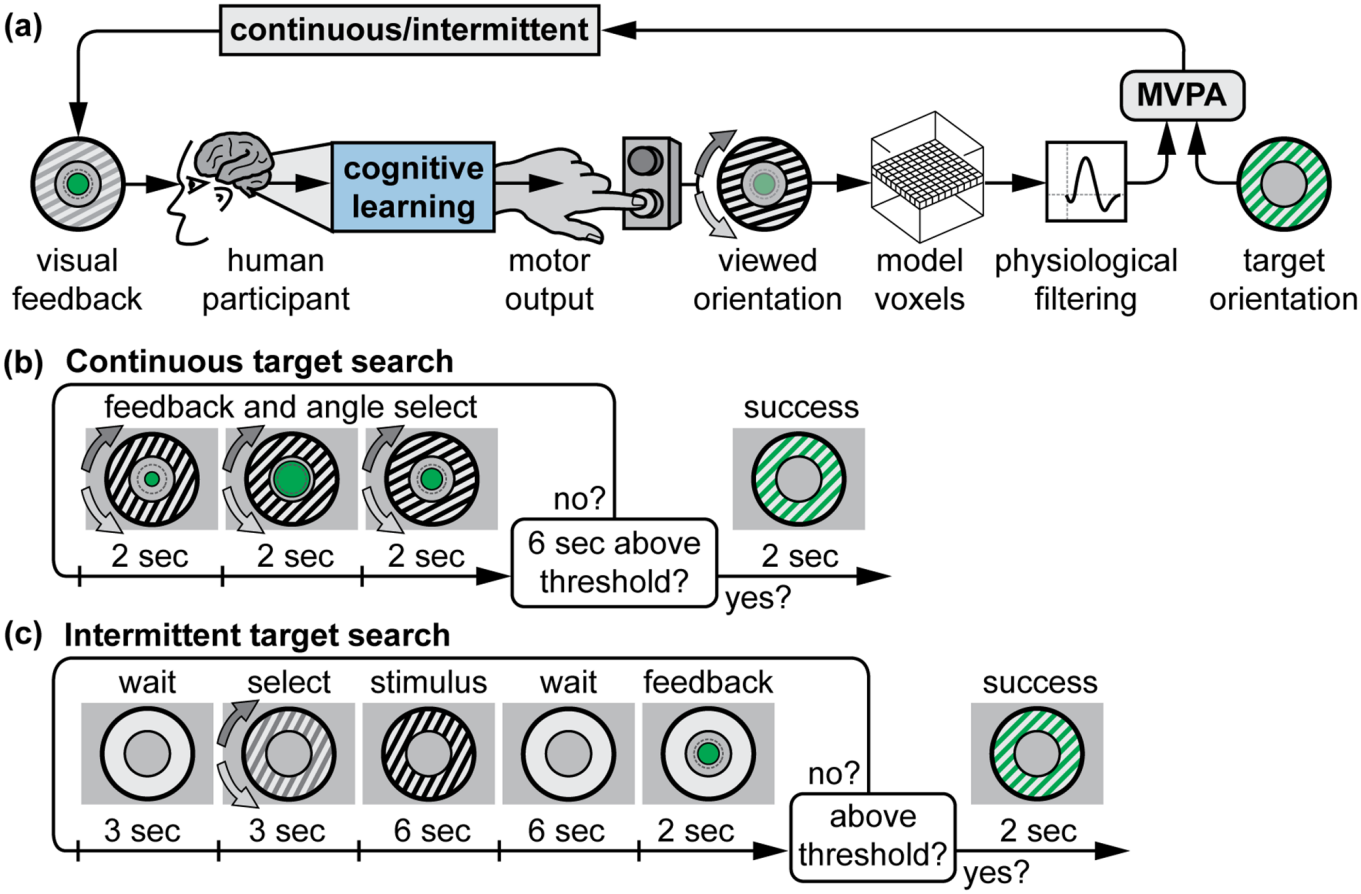
Cognitive learning of neurofeedback signals. **(A)** In this simulation, participants influence simulated neural activity by using a motor strategy to stimulate our model of V1. Button presses rotate a grating, which then induces activity in the V1 model. After physiological filtering, the resulting volume is decoded based on a target orientation and presented as visual feedback either continuously or intermittently. **(B)** For continuous feedback target searches, participants are able to continuously update the grating orientation, and the feedback signal updates every 2 seconds. **(C)** For intermittent feedback target searches, participants select one orientation each trial and receive summarized feedback at the end of each trial.

We created a real-time feedback environment where human participants made cognitive decisions to maximize a simulated neurofeedback signal. Mental imagery is the most commonly used cognitive strategy in neurofeedback [18], but is difficult to quantify, varies between subjects, and cannot be reported on a second-to-second basis. Here we used measurable overt motor output as a proxy for mental strategies. Human participants used one of two buttons to rotate a grating image clockwise or counter-clockwise (Fig 3A). This grating image directly stimulated our V1 model based on its orientation. During each trial, participants were instructed to find a target orientation between 0° and 180° by rotating the grating. Participants found these targets by interpreting a decoded fMRI-like feedback signal indicating the probability that the grating was aligned with the target orientation. The signal was decoded using an 8-way sparse logistic regression classifier [31] trained on simulated data from each of the eight tuned orientations of our V1 model (Fig 2A). In this way, we operationalized a mental imagery strategy as a simple motor decision that influenced a model of V1 neural activity. In contrast to more implicit neurofeedback learning experiments [25, 29], the feedback received by participants was directly related to the physical properties of the grating stimulus which participants viewed. Thus, it eliminated any ambiguity about the relationship between their strategy choice and the feedback signal other than temporal delays and blurs. We address the additional complexity of implicit learning in our automatic learning simulations, which did not explicitly use the grating stimulus to generate feedback signals.

Participants were assigned into continuous and intermittent feedback timing groups (Fig 3B and 3C) and exposed to all of the simulated physiological filters (Fig 1E) to examine how these modified or impaired participants’ cognitive strategies. In addition to our hypothesis that the hemodynamic response should be more difficult to learn than isolated delay or blur responses, we also hypothesized that participants in the intermittent feedback group would find targets faster since this feedback timing makes the credit-assignment problem easier by accounting for the hemodynamic delay.

### Continuous feedback is superior to intermittent feedback for simple cognitive strategies

**Fig 4:**
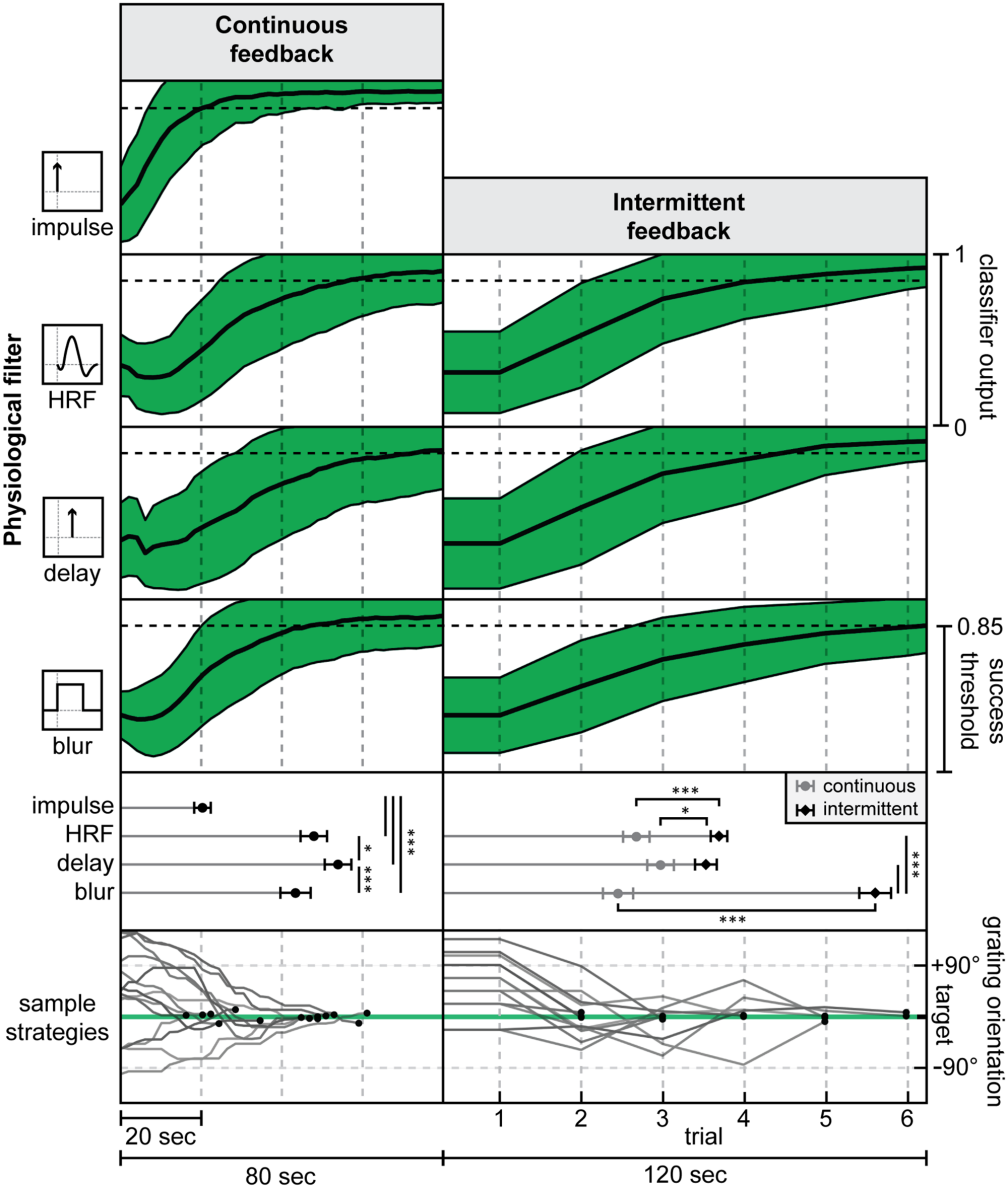
Cognitive learning curves. Learning curves were constructed by combining all target searches over all participants for each physiological filter. Plots in green show the mean classifier output (+/-s.d.) over the course of each target search. Because a new target was selected as soon as the current target was reached, all time points after success were simulated using randomly generated volumes at the target +/-5°. For means (+/-s.e.) of time to target for continuous (circles) and intermittent (diamonds) feedback, stars indicate significant differences at p<0.05 (*) and p<0.001 (***). In the sample strategy plots, the grating orientation for all HRF target searches from one participant (continuous: participant #3; intermittent: participant #9) are plotted relative to the target, with black circles indicating the time at which the target was reached.

In our cognitive simulation, we were able to create a one-dimensional cognitive strategy (selection of grating orientation) that could be quickly understood by participants. In this setting we found that in contrast to our hypothesis, the continuous feedback group was able to find targets faster than the intermittent feedback group for the HRF, blur, and delay physiological filters (Fig 4). The largest difference was found in the blur condition (t_(36)_ = 11.06, p<0.0001), with a large difference also found in the HRF condition (t_(36)_ = 4.564, p<0.0001). The delay condition was the most similar between continuous and intermittent conditions, but continuous feedback was still significantly better (t_(36)_ = 2.363, p = 0.0237).

These results show that if participants can directly influence the feedback signal through simple action choices, they are able to rapidly learn its dynamics and can perform better than those receiving intermittent feedback. This suggests that feedback of physiological responses that delay or blur the underlying neural activity can be successfully learned through cognitive means with a continuous feedback signal, as long as an effective strategy (i.e. a specific, well-defined behavior) is known to the participant. It is important to note that these conclusions may not generalize to explicit imagery tasks where many and more complex cognitive strategies could be used, when there are interdependencies between these strategies, or when the signal to noise ratio (SNR) of the neural signal is poor. For instance, in a study concluding that intermittent feedback was superior [19], the cognitive strategies employed by participants may not have been effective, and automatic processing of the intermittent feedback may have occurred in parallel to enable superior self-regulation compared to continuous feedback.

### Physiological signal properties impact neurofeedback performance

Physiological filters also affect learning rate within each of the continuous and intermittent feedback conditions (Fig 4). In the case of continuous feedback, the impulse condition was found to be significantly easier to learn than the HRF (Tukey’s HSD, p<0.0001), delay (p<0.0001), and blur conditions (p<0.0001). The pure delay condition was significantly more difficult to learn than both blur (p<0.0001) and HRF (p = 0.0305). We also found a trending, non-significant effect suggesting that blur is slightly easier than HRF (p = 0.153). For continuous feedback, an instantaneous neurofeedback signal derived from an impulse response filter was the easiest signal to learn. The HRF physiological response was also significantly more difficult to learn than the impulse response. The isolated delay and blur responses put the HRF result in context: a pure delay was even more difficult to learn than HRF or blur. The increased difficulty of the delay physiological response conflicts with our hypothesis: we expected the HRF, which is a combination of delay and blur, to be the most difficult to learn. However, these results can be explained by the amount and timing of the contingent information received with each physiological response. For the impulse, participants receive 100% of the feedback information within 2 seconds. For blur, participants receive 20% of this information within 2 seconds, with the remainder of the information accumulating over the subsequent 10 seconds. The HRF filter provides no immediate information, but instead provides smeared and sluggish information over the next 6 seconds. The delay response gives no information at all until 6 seconds after the underlying neural activity has occurred. To summarize, it appears that the sooner that any neurofeedback information (even from partial or degraded signals) reaches the participant, the sooner they are able to adjust their cognitive strategy to find the target more quickly.

For intermittent feedback (Fig 4, right), the blur condition resulted in significantly worse performance compared to HRF (Tukey’s HSD, p<0.0001) and delay (p<0.0001). There was no significant difference between the HRF and delay conditions in the intermittent feedback case (p = 0.672). An artefact in the intermittent feedback calculation caused this sharp drop in performance with the blur response. The 6-sec stimulus period and subsequent 6-sec feedback calculation period of the intermittent feedback paradigm are well-matched to the HRF and delay responses, but not to the 10-sec moving average of the blur response. For the same grating orientation stimulus, the blurred response resulted in a lower peak classifier output on average during the feedback calculation period. This meant that participants needed to be more accurate to reach the feedback signal success threshold. This shows that neurofeedback performance can be degraded if intermittent feedback timing and calculation are not well-matched to the physiological response properties of the signal being measured.

### Automatic neurofeedback learning: Strengthening neural patterns using reinforcement of spontaneous activity

While we found continuous feedback to be superior to intermittent feedback for cognitive strategies, this need not be true for all fMRI neurofeedback studies. A major drawback of our cognitive learning experiments is that they assume a static relationship between stimulus and brain activity. They effectively bypass the complexity of the brain by providing a direct pathway between stimulus and feedback signal in the form of a visually-presented grating. This is equivalent to a participant having a set of mental strategies that consistently map onto patterns of neural activity and that can be recalled as easily as pressing a button. This is clearly unrealistic if the trained neural circuit has no clear associated cognitive strategy or participants need to be kept unaware of the experimental manipulation. In these cases, implicit or automatic neurofeedback strategies must be used.

**Fig 5:**
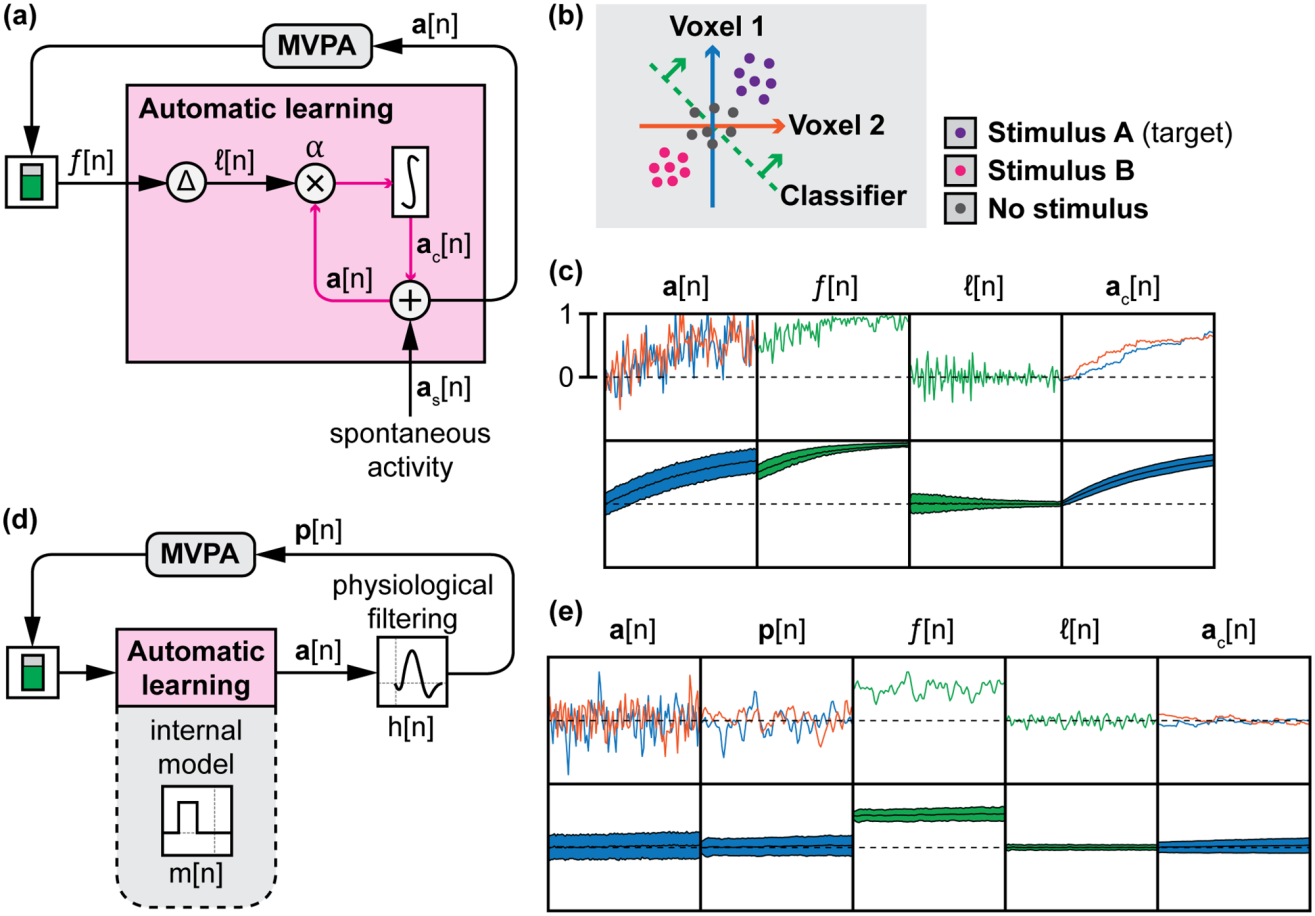
Automatic learning of neurofeedback signals. **(A)** Reinforcement learning is used to shape neural activity in the absence of delay. **(B)** A simple two-voxel neural model is used to demonstrate how our model of automatic learning can learn a pattern of neural activity associated with a stimulus. **(C)** Without delays, our model is able to learn the desired pattern of neural activity. The top row shows example data from one trial, while the filled plots on the bottom row show the mean and 50% confidence interval (CI) for each signal at each time point, averaged over 1000 simulated trials. **(D)** If significant physiological delays exist, then an internal model must exist to hold in memory underlying neural activity before it can be reinforced by the delayed feedback signal. **(E)** If no internal model exists, learning of neural activity filtered by the HRF does not occur.

If no voluntary action or external stimulus influences neural activity, then we are faced with the challenge of controlling a neural signal that is driven by unconstrained cognitive states (e.g., mind-wandering) along with a contribution from spontaneous neural activity. Over time, participants should be conditioned to elicit the desired pattern of activation whenever they anticipate a neurofeedback signal or when presented a conditioned cue. We cannot examine this phenomenon with human participants unless we use a real neural signal. Instead, we constructed a computational model that started with purely random, spontaneous activity patterns and learned to elicit desired activity through reinforcement provided by an MVPA classifier that was trained to recognize a desired pattern (i.e. simulated activity patterns corresponding to a stimulus). This model of automatic learning (Fig 5) followed a basic reinforcement learning structure [32]: changes in a feedback signal are used to reward or punish spontaneous neural activity, which is accumulated (integrated) over time.

Consider a vector of activity **a**[*n*] at time point (TR) *n*, which is a combination of spontaneous activity **a**_*s*_[*n*] and learned (conditioned) activity **a**_*c*_[*n*]. Let **a**_*s*_[*n*] = *N* ∼ (0, *σ*_*s*_), where spontaneous activity in each voxel is independent (we will later introduce spatial correlations). Given a matrix of classifier weights $W_{N_{voxel}xN_{class}}$ and a learning rate *α*, we can ‘learn’ the next conditioned activity, **a**_*c*_[*n*+1] by observing the change in classifier output due to the current activity pattern. Defining *f*[*n*] as the classifier output for the target (feedback signal) and *l*[*n*] as the learning signal:

$$\begin{array}{c} \begin{array}{cc} & a\left[n\right]=a_{s}\left[n\right]+a_{c}\left[n\right]\\ & f\left[n\right]=softmax(W^{T}a\left[n\right])\\ & f\left[n\right]=f{\left[n\right]}_{i=target}\\ & l[n]=f[n]-f[n-1]\\ & a_{c}\left[n+1\right]=\alpha \cdot l\left[n\right]\cdot a\left[n\right] \end{array} \end{array}$$ (1)

Where *softmax*(⋅) = *e*^(⋅)^/Σ*e*^(⋅)^ ensures that the feedback signal represents the likelihood of the target class being observed. In this example, feedback is instantaneous to the underlying neural activity (e.g. the physiological response is an impulse), and spontaneous activity can easily be rewarded because the feedback and underlying neural activity are occurring at the same time.

**Fig 6:**
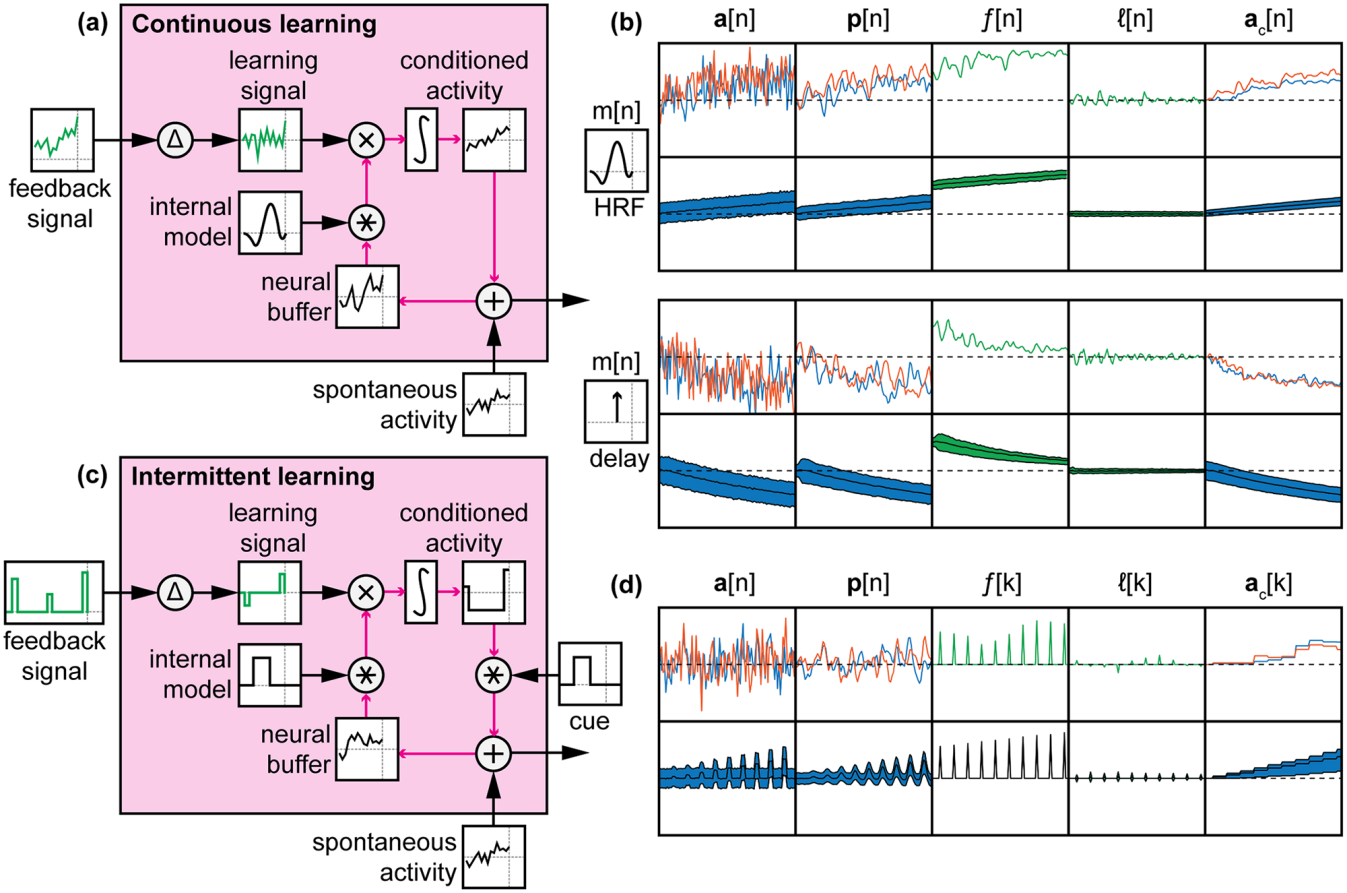
Automatic learning of continuous versus intermittent neurofeedback signals. **(A)** For continuous learning, a buffer of neural activity is kept in memory and filtered by the internal model. **(B)** In continuous learning, learning can occur when the internal model accurately matches the underlying physiological response. However, with a delay internal model, anti-learning occurs when the underlying neural activity is filtered by the HRF. **(C)** For intermittent learning, a cue is used to gate the conditioned activity. **(D)** Intermittent feedback allows trial-by-trial learning of underlying activity that is filtered by the HRF.

Our next step was to test this automatic feedback learning structure in a simple simulation: a two-voxel brain with two classes of stimuli and a classifier weighting $W=\left[\begin{array}{ll} 1 & -1\\ 1 & -1 \end{array}\right]$ (Fig 5B). We used a learning rate *α* = 1 and spontaneous activity *σ*_*n*_ = 0.25. Using Eq (1), we expectedly found that given a desired activity pattern, the desired activity was elicited (Fig 5C). However, we hypothesized that this simple learning rule would be unable to learn the underlying neural activity if the feedback signal was filtered by some sort of physiological response such as the HRF before being measured and presented as feedback (Fig 5D). Indeed, we found that this physiological filtering impairs learning (Fig 5E). We are aware of models that exist for automatic learning of near-instantaneous electrophysiological signals [33], but not for signals with a time delay similar to the HRF. For these longer delays, we posit that an internal model of the relationship between underlying neural activity and feedback signal must exist to solve the credit-assignment problem by holding in memory the underlying neural activity until the feedback signal can be presented (Figs 5D and 6A). This internal model is purely temporal and has no spatial awareness. All voxel activities are rewarded or punished equally by the feedback signal at specific time points in the past, where the weighting of time points is defined by the internal model. This is analogous to a sliding window memory of whole-brain neural activity [17] that a delayed feedback signal is able to reward or punish.

To simulate this internal model, we must augment Eq (1) with *h*[*n*] as the delayed or blurred physiological response, *m*[*n*] as the internal memory model, and **p**[*n*] as the delayed or blurred physiological activity:

$$\begin{array}{c} \begin{array}{cc} & p[n]=a[n]*h[n]\\ & f\left[n\right]=softmax(W^{T}p\left[n\right])\\ & a_{c}\left[n+1\right]=\alpha \cdot l\left[n\right]\cdot \left(a\left[n\right]*m\left[n\right]\right) \end{array} \end{array}$$ (2)

If we assume a maximum length *L* of the physiological response and internal model, we can expand Eq (2) as follows:

$$\begin{array}{l} a_{c}\left[n+1\right]=\alpha \left(f\left[n\right]-f\left[n-1\right]\right)\cdot \sum_{n=-L}^{0}\left(a\left[n\right]m\left[-n\right]\right)\\ f\left[n\right]=softmax{\left(W^{T}\sum_{n=-L}^{0}\left(a\left[n\right]h\left[-n\right]\right)\right)}_{i=target} \end{array}$$ (3)

Although the *softmax* calculation prevents further meaningful simplification of these equations, we can see from Eq (3) that matching *m*[*n*] to *h*[*n*] should help reduce the learning problem toward a summation of impulse learning problems (e.g. those in Eq (1)). Indeed, if we include an internal model that matches the underlying physiological response, such as an HRF internal model for the HRF physiological reponse, we see that learning can now occur (Fig 6B, top). However, if the internal model is not perfectly matched to the actual neural response, such as a delay internal model, we see that it is possible for anti-learning to occur: the opposite pattern of desired activity is learned (Fig 6B, bottom). This result is of critical importance because participants’ internal models cannot be verified and because a ‘pure delay’ model is typically one of the verbal instructions given to participants to describe the hemodynamic lag [18]. Therefore, with continuous feedback, there is risk that an incorrect internal model will be generated, leading to poor learning or even anti-learning of the neurofeedback signal.

So far, we have only considered the problem of continuous feedback. We initially hypothesized that intermittent feedback would be superior to continuous feedback, and although this was not the case for cognitive strategies, we will now examine intermittent feedback in the context of automatic learning. For intermittent feedback, learning during each trial *k* with cue period *n*_*c*,1_ ≤ *n* ≤ *n*_*c*,2_ and subsequent pre-feedback wait period *n*_*w*,1_ ≤ *n* ≤ *n*_*w*,2_ takes the following feedback structure:

$$\begin{array}{c} \begin{array}{cc} & a\left[k\right]=\frac{1}{n_{c,2}-n_{c,1}+1}\sum_{n=n_{c,1}}^{n_{c,2}}(a_{s}\left[n\right]+a_{c}\left[n\right])\\ & f\left[k\right]=\frac{1}{n_{w,2}-n_{w,1}+1}\sum_{n=n_{w,1}}^{n_{w,2}}f\left[n\right]\\ & l[k]=f[k]-f[k-1]\\ & a_{c}\left[k+1\right]=\alpha \cdot l\left[k\right]\cdot a\left[k\right] \end{array} \end{array}$$ (4)

Using this intermittent feedback structure (Fig 6C), the learning problem in Eq (4) is roughly reduced to the impulse learning problem of Eq (1), albeit on a slower time scale. Using the HRF physiological response in our automated learning simulations, a cue period of 3 TRs, and a subsequent wait period of 3 TRs, we found that intermittent feedback also led to successful learning (Fig 6D).

### Activating simulated visual cortex using reinforcement of spontaneous neural activity

**Fig 7:**
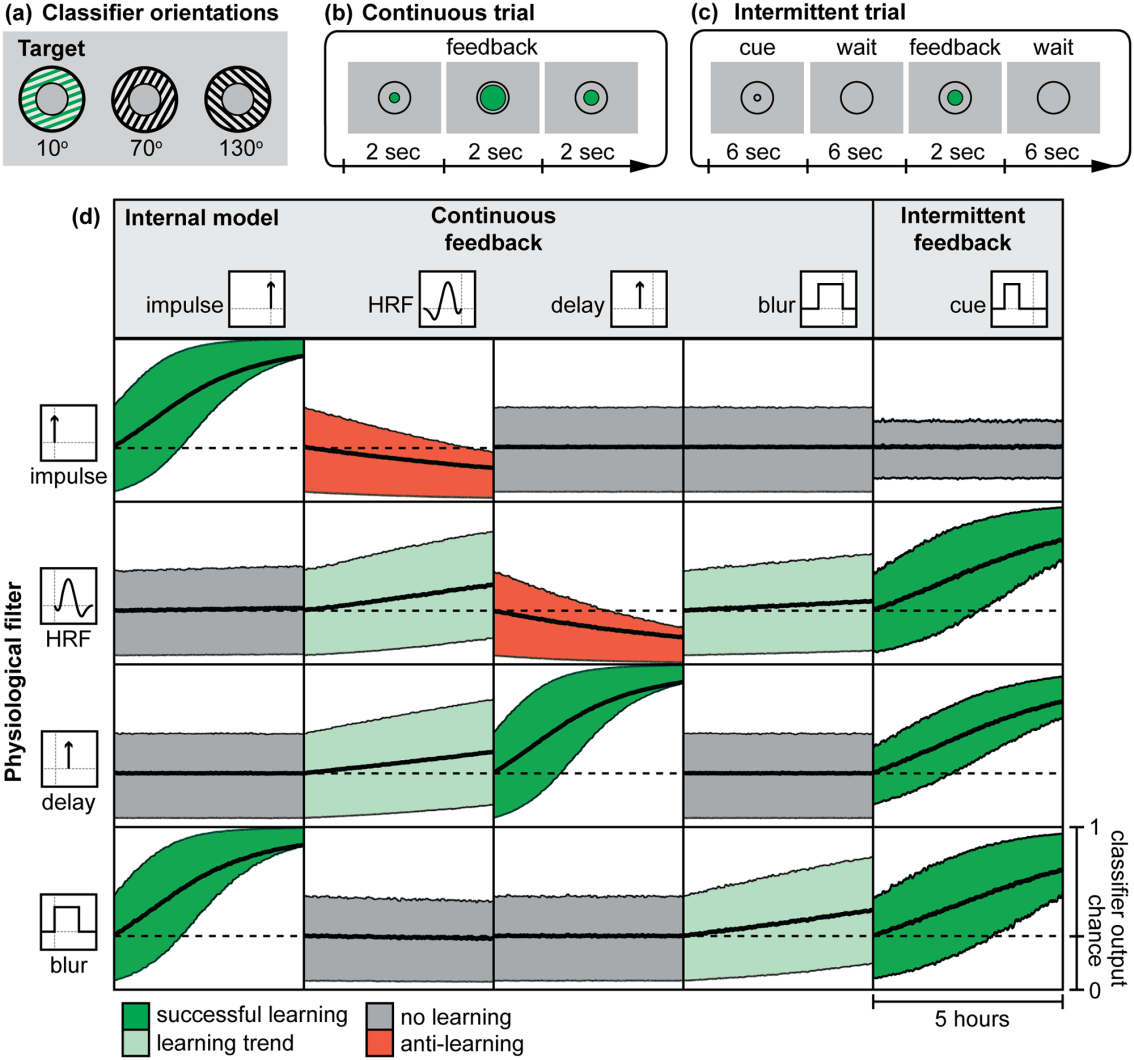
Automatic learning of grating patterns in simulated V1. **(A)** A 3-way sparse logistic regression classifier was used, with feedback provided from the 10° classifier output. **(B)** For continuous trials, feedback was provided to the learning model every 2 seconds. **(C)** Intermittent trials followed a cue-wait-feedback structure. **(D)** Learning curves were generated by averaging the classifier output at each time point over 1000 simulated participants for each condition. Filled plots indicate the mean and 50% confidence interval (CI) for each time point, which was further filtered using a 200-sec moving average filter (about 1% of the total time course) for graphical presentation purposes. Chance is indicated at 0.33. Plots are colored according to feedback success.

Given that our automatic learning model worked to elicit a desired pattern of activity in a simple two-voxel model, our next goal was to train activation patterns in our full V1 model without directly stimulating it with grating images (i.e. a simulation of the experiment performed by Shibata et al. (2011) [25]). Using the same neural model as the cognitive experiment (which contains spatial noise correlations typical of fMRI), we constructed a 3-way sparse logistic regression classifier (Fig 7A) and aimed to elicit the pattern of activity associated with a grating oriented at 10°. Our comparison of continuous versus intermittent feedback was similar to the cognitive experiment, with continuous feedback provided every 2 seconds (Fig 7B), and a cue replacing the direct stimulus during intermittent trials (Fig 7C). We then aimed to test how different internal models affected automatic learning. Specifically, we addressed whether learning could occur with different delayed or blurred underlying physiological responses, and if so, how accurate did the internal model need to be? In addition to a general hypothesis that intermittent feedback would be superior, we hypothesized that for continuous feedback, matched filter-model combinations (e.g. HRF-HRF) would produce the best learning, whereas incongruent filter-model combinations (e.g. HRF-delay) would produce the least learning.

### Automatic learning of neurofeedback signals requires an accurate internal model

Our automatic learning simulations show that successful learning in the absence of cognitive strategies depends on the underlying physiological response and the internal model used to interpret that temporal response (Fig 7D). For example, for the HRF physiological response, successful learning was found with a cue model. Moderate learning was found with HRF and blur models, with HRF leading to quicker learning than blur. No learning was found with an impulse model, and a delay model resulted in anti-learning. These results align with our simple two-voxel model (Fig 6). This confirms that increased dimensionality and moderate spatial noise correlation do not have an effect on the working principle of our automatic learning model.

Contrary to our hypothesis, we found that with a continuous feedback signal, matched filter-model combinations only led to successful learning for impulse-impulse and delay-delay combinations. The incongruent blur-impulse filter also showed successful learning and was superior to the matched blur-blur model. We believe this can be explained by the temporal dependency of the system: the learned signal is integrated over time, so successful spontaneous activity should be captured and reinforced as soon as possible. With the impulse internal model, as soon as successful activity occurs, it is captured by the blur filter and is immediately reinforced. With a blur internal model, this successful activity is only reinforced at 20% strength (averaged across the previous 5 TRs, or 10 sec of simulated neural data), and becomes lost in the noise. Another temporal relationship may also explain why a delay internal model does not result in learning of the blur filter, whereas an impulse model does. Both of these internal models sample one time point from the blur filter, and all 5 TRs (10 seconds) are weighted equally. However, our model only learns when there is a change in the feedback signal. When successful activity occurs, the change in feedback signal is immediately captured by the blur physiological response filter and reflected as a change in the feedback signal that an impulse internal model can learn from. If a delay internal model is used instead, there is no change in feedback signal at the 3 TR delay, and thus no learning occurs.

For intermittent feedback, we see successful learning for the HRF, delay, and blur filters. Indeed, this feedback schedule has been successful for automatic learning of fMRI acivity patterns [25, 28]. These results suggests that the same intermittent feedback schedule can be used to learn a variety of physiological responses as long as the neural activity in the cue period is reliably recorded during the wait period.

### Automatic learning of spatial patterns depends on spontaneous activity correlations

**Fig 8:**
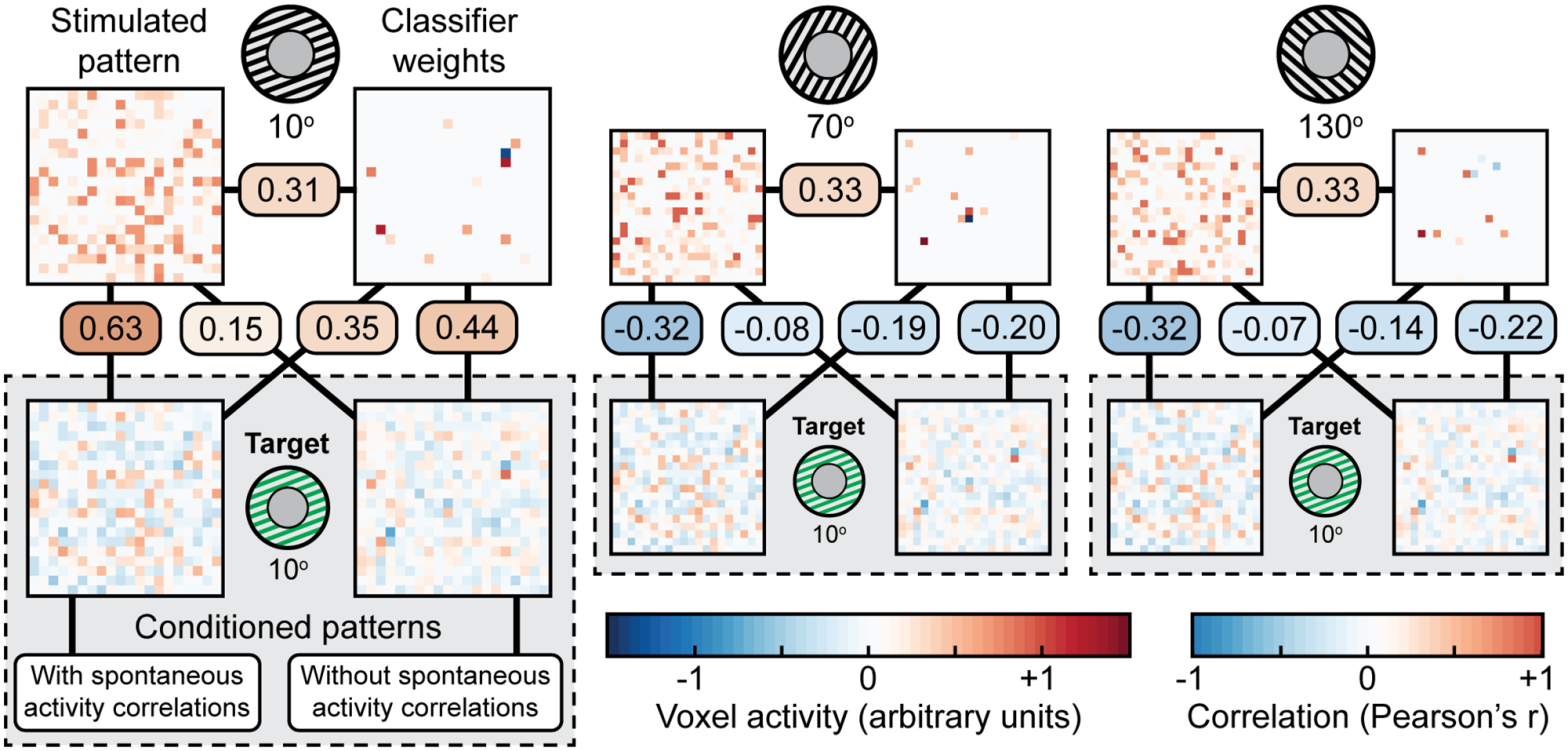
Conditioned patterns of activity with and without spontaneous activity correlations. True orientation-associated patterns, classifier weight maps, and conditioned activity patterns were projected onto a 2D surface. Patterns of activity were distributed across voxels and were not dominated by one or two voxels. For each of the three classifier orientations (Fig 7A), the true underlying pattern associated with the stimulus is shown (without spontaneous activity added), as well as its corresponding classifier weight map. 1000 patterns were conditioned to purely random Gaussian noise (‘without spontaneous activity correlations’), while 1000 patterns were conditioned with a mixture of Gaussian noise and a random orientation signal (‘with spontaneous activity correlations’). Feedback was provided from the 10° classifier output for both types of conditioning. One example of each type of conditioned pattern is shown. The displayed correlations are the mean correlation across all 1000 patterns.

While our model was able to learn to achieve a large classifier output, it is possible that this occurred simply due to activity in one or two voxels. To address this concern, we performed an additional simulation with visualizable activity patterns (Fig 8). The 200 tuned voxels and 3 classifier weight maps from our V1 model were randomly placed onto a 20x20 (400-voxel) surface. Conditioning was performed using the hrf-cue filter-model combination from Fig 7D, as is common in decoded neurofeedback experiments [25]. Over 1000 simulated participants using the same noise parameters as Fig 7 (e.g. without spontaneous activity correlations), we found that the classifier output was indeed driven by a distributed pattern of activity. However, this activity correlated more strongly with the classifier weights associated with the target orientation (*r* = 0.44) than the true orientation-associated pattern (*r* = 0.15). This pattern of correlations was also found in the two non-target orientations: larger magnitude correlations with classifier weights (*r* = −0.20, −0.22) relative to the true orientation patterns (*r* = −0.08, −0.07). These negative correlations indicate that the patterns associated with non-target orientations are punished, which is consistent with the *softmax* operation of the classifier capturing information from all three classifier weight maps.

Weak correlations with the true orientation patterns are likely related to the sparsity [31] of the classifier: the classifier does not select all voxels associated with the true orientation pattern, so how could the model learn activity in these unselected voxels? True patterns of spontaneous activity in V1 are not truly random: they tend to contain information associated with visual attributes [34]. If orientation signals are present in the spontaneous activity, then our model should be able to learn activity in voxels not selected by the classifier. To test this hypothesis, we introduced a noise model that was a half-and-half mixture of random Gaussian noise and a random orientation signal between 0° and 180°. This random orientation signal ensured that while visual attributes were included in the spontaneous activity, it did not have any bias toward the target orientation. Using the same conditioning procedure as before, we found that this noise model resulted in conditioned patterns that had much stronger correlations with the true target pattern (*r* = 0.63) and weakened correlations with the target classifier weight map (*r* = 0.35) (Fig 8).

## Discussion

We simulated fMRI activity in visual cortex to determine how feedback timing affects learning of neurofeedback signals with different physiological responses and self-regulation strategies. Our first experiment had human participants find hidden visual targets (oriented gratings) using only simulated neural activity as a feedback learning signal. We used four physiological filters in this experiment: instantaneous feedback, the canonical HRF of fMRI, and isolated delay and blur filters. One group was exposed to continuous feedback, while another saw intermittent feedback. In a second experiment, we fully simulated the automatic learning of neurofeedback signals, examining how continuous and intermittent feedback could be used to induce a pattern of activity in simulated visual cortex without stimulus presentation. We found that continuous feedback was best for cognitive strategies, whereas intermittent feedback was better for automatic learning. For cognitive learning of neurofeedback, we found that all properties of hemodynamics impair neurofeedback learning, but the delay of the continuous feedback signal affects learning more than the blur. For automatic learning, we found that intermittent feedback was able to absorb differences in hemodynamic properties to facilitate learning, whereas a precise and accurate model of the hemodynamic response was necessary for learning with continuous feedback.

Our analyses isolated cognitive and automatic learning of a simulated neurofeedback signal under ideal conditions. In practice, neurofeedback experiments are often underspecified, and may rely upon some combination of both learning techniques [8]. Because of the interaction between feedback timing and learning technique, our results suggest that either cognitive or automatic learning strategies should be chosen before feedback timing is scheduled in an fMRI neurofeedback experiment. Furthermore, while the relative effects of blur and delay shown in our cognitive results apply to all neurofeedback studies with a prescribed cognitive strategy, the absolute performance we reported (e.g., time-to-target in a search for a hidden target neural activity pattern) does not apply to the majority of fMRI neurofeedback studies because of the simple, unidimensional nature of our cognitive strategy and the arbitrary SNR that was used. Increasing noise and allowing for more complex, multidimensional strategies could drastically increase time-to-target in our simulation. The static relationship between stimulus and brain activity was a further simplification that may not hold true when the ‘stimulus’ is a complex cognitive strategy chosen by a participant. Importantly, these pitfalls do not apply to automatic processing: strategy is not chosen by the participant, and noise due to spontaneous activity is displayed as useful information in the feedback signal. Therefore, unless a simple, known cognitive strategy will reliably elicit the desired neural activity, our results suggest that automatic processing is more likely to result in successful learning of fMRI neurofeedback signals.

Our automatic learning model answers the question: given an internal model of the temporal dynamics of the feedback signal, how is the feedback signal learned? It does not attempt to explain how this internal model may be generated or adapted over time. While the feedback/wait/cue structure of intermittent feedback is a plausible mechanism to create a stable internal model that is agnostic to the actual temporal characteristics of the underlying physiological response, it is unclear how an internal model would be created for a continuous feedback signal. For example, given verbal instructions about the blur or delay of the feedback signal, their translation into a filter applied to an internal memory buffer of neural activity is unknown. In the absence of instruction, animals effectively use a short time scale, near-impulse model to self-regulate continuous electrophysiological signals (For a detailed model involving spiking neurons, see Legenstein et al. (2008) [33]). For longer time scales, it is widely accepted that conditioning can occur as long as the delay to reinforcement is predictable [35]. However, without a predictable cue structure, a pessimistic viewpoint would argue that the impulse model is used by default for continuous feedback signals, which is ineffective for learning feedback signals that are significantly delayed or blurred.

There is evidence to show that a neural representation of time exists to store neural activity in memory [17]. This temporal representation becomes less accurate the longer the delay, but nevertheless provides a mechanism for a continous-time internal model. The problem is then still: which time delay does the brain choose to reinforce? The difficulty of creating an appropriate internal model may explain the inability for some participants to gain control over continuous fMRI neurofeedback signals, and could therefore underlie the “non-responder” challenge in human neurofeedback [10]. Our simulation results are inconsistent with the results of Ramot et al. (2016) [36], who used continuous feedback of the hemodynamic response to shape neural activity in the absence of awareness of the meaning of the feedback signal. It would appear that either participants were able to generate a working internal model of the temporal characteristics of the feedback signal (e.g. the blur or HRF internal models applied to the HRF physiological response in Fig 7D) in the absence of external cues, or these results were simply obtained by chance without any true neuromodulation due to the feedback signal. Another pitfall of using continuous feedback in automatic learning is that an incorrect internal model can result in anti-learning. For example, the impulse-HRF and HRF-delay models end up worse than chance (Fig 7D). Verbal instructions may help participants tune their internal models, but given that one of the common instructions (a 6-second delay internal model) resulted in anti-learning in our simulation, this seems risky compared to simply scheduling feedback intermittently.

Our automatic learning simulations also shed light on the debate regarding how patterns of activity can be learned in a high-dimensional voxel space [37, 38]. Huang (2016) argues that because participants do not have cognitive access to the voxel space, the dimensionality of this space is too large for participants to reliably discover an effective cognitive strategy to activate the desired pattern. Shibata et al. (2016) counter-argue that the dimensionality of the search space is actually much smaller than the voxel space would suggest due to correlations in V1 activity. We provide an alternative explanation to both arguments. First, cognitive access to the voxel space is not required since automatic learning circuits can reward the underlying neural activity without participant awareness. Second, a global (whole-brain) reward signal allows complex activity patterns to be conditioned, even with high-dimensional spontaneous neural activity patterns.

The neurofeedback simulation framework reported here is intended as a proof of concept: we started with a simplified model of V1 for clarity. Looking forward, this model has two major limitations. First, our noise model did not include any measurement noise, so all simulated activity was a result of spontaneous activity and not error in the simulated measurement. Measurement noise would almost certainly have a negative impact on learning, but the ratio of signal/spontaneous/measurement noise in fMRI is difficult to quantify [39] so we chose to simplify our model and leave open the potential to improve its specificity in future iterations. Our noise was also Gaussian with local spatial correlation. This noise model is not sophisticated enough to capture the true complexity of visual cortex: we know that correlational structures exist within spontaneous activity in V1, and that these patterns tend to correlate with basic visual attributes [34]. We also know that using neurofeedback signals that match patterns of spontaneous activity are easier to learn [40]. Therefore, adding a more realistic correlational structure to our noise model should not affect the qualitative relationship between physiological responses and internal models, but rather it should show that patterns that commonly occur spontaneously will be easier to learn through neurofeedback. Indeed, when we added random visual attributes to our spontaneous activity model (Fig 8), we found that conditioned activity patterns more closely matched the target orientation pattern compared to conditioning with completely random spontaneous activity. Second, our spatial capture model was based on meta-parameters from prior work [4]. An advanced spatial capture model, such as one recently developed by Kriegeskorte and Diedrichsen (2016) [41], could be used as a basis for the simulated neurofeedback signal. This capture model shows that shifts in voxel sampling over finer-grained neural activity can bias the recorded representational structure of stimuli. Such a model could help determine whether neurofeedback is feasible given a specific neuroimaging method and targeted neural pattern.

Overall, our model demonstrated an important contrast between cognitive and automatic neurofeedback strategies, suggesting that different self-regulation mechanisms prefer different feedback schedules. These findings have significant implications for any experiment involving the feedback of delayed or blurred signals, and have direct applications for the design of neurofeedback schedules and instructed strategies for future fMRI neurofeedback experiments. Furthermore, the simulation framework provides a basis to evaluate and optimize the design of such experiments before they are performed with real neuroimaging data.

## Methods

### Ethics statement

This study was approved by the University of Texas Institutional Review Board. Written informed consent was obtained from the participants.

### Participants

Forty-eight healthy participants (27 female; average age 20.34 years, SD = 3.08) were recruited for the experiment. Participants were randomly assigned into 2 groups: 24 received continuous feedback and 24 received intermittent feedback.

### Apparatus

Participants were seated at a computer (21.5” iMac) and used the arrow keys on a standard keyboard to perform the experiment. Data were sampled and displayed at 60 Hz using Matlab and Psychtoolbox 3 on Mac OS X. No neuroimaging apparatus was required.

### Model parameters: Voxel-based V1 captured by simulated fMRI

Our model of V1 (Fig 2a) is based on meta-parameters extracted from Kamitani and Tong (2005) [4]: a 1000-voxel cube of 3x3x3mm voxels, with 20% of voxels tuned to grating orientation (2.5% to each of 8 orientations) and 80% of voxels untuned. The tuning curve for each voxel is a decaying exponential, with full output (arbitrary units) at the tuned orientation, decaying to 1/16 output at the orthogonal orientation. Underlying neural activity is generated according to the orientation of the stimulus and the corresponding voxel tunings. Spatially correlated spontaneous activity (Gaussian random field with 5mm kernel) is added to this tuned activity and the result is convolved in time with a physiological response filter.

The four physiological responses (Fig 1E) are sampled at 0.5Hz, corresponding to the 2 second time repetition (TR) of standard fMRI capture. Therefore, the impulse samples one TR of activity with no delay, the delay samples one TR of activity with a 3-TR delay, and the blur averages over the previous 5 TRs. The HRF filter is a weighted average of the previous 15 TRs of activity, according to the canonical fMRI BOLD response. All filters were normalized such that the sum of their weights equalled one.

### Model parameters: Cognitive learning

The classifier used to decode cognitively-elicited simulated fMRI patterns in real-time (Fig 2B) used a sparse logistic regression classifier [31] because this technique has been successfully applied to fMRI data from V1 [24]. To train the classifier, we simulated 800 training examples (100 each of the 8 tuned orientations: 0°, 22.5°, …, 157.5°) using a voxelwise signal-to-noise ratio (SNR) of 2 (tuning curve peak = 1; spontaneous activity *σ*_*n*_ = 0.5). These parameters were chosen to approximate the results from a two-hour-long pattern localizer experiment [4].

We generated simulated brain activity online using the predetermined grating orientation-voxel activation relationship. For each time repetition (TR = 2 seconds), the mean grating orientation during that period was input to the model. Through piloting, we found that with a realistic SNR of 2, participants often reached targets by chance rather than through interpretation of the feedback signal. We therefore increased the SNR of all voxels (tuned and untuned) to 10 (spontaneous activity *σ*_*n*_ = 0.1). This increased the reliability of the feedback signal for participants so that we could more readily examine the temporal characteristics of the physiological signals.

The simulated neurofeedback signal was calculated using a weighting of the eight decoded orientation probabilities from the classifier. Specifically, the feedback signal was calculated by the dot product of the vector of classifier probabilities and a tuning curve, where the tuning curve was a decaying exponential circularly shifted to align its peak with the target orientation. This resulted in a smooth, gradually increasing feedback signal throughout the stimulus orientation range, instead of one that remained low and then peaked sharply at the target orientation. For example, if the classifier was 100% confident that the grating was 22.5° away from the target, the classifier would output a 0% probability for the target orientation, even though the participant was near the target. By using the dot product of the tuning curve and the raw classifier output for all orientations, the simulated neurofeedback signal for a grating 22.5° away from the target is 50% (instead of 0% if only the classifier output for the target orientation was used). If the classifier was 100% confident the grating was 45° away from the target, this dot product would instead output 25%, and so on. Importantly, the scalar output of this dot product always lies between 0 and 1, so it can easily be displayed on a thermometer without additional scaling.

To determine whether participants had reached the target, a threshold of 85% classifier output at the target was selected because this corresponded to roughly 5–10 degrees of grating orientation error from the target (depending on noise) and was feasible for participants to reach during piloting of the experiment. If the average classifier output over 3 consecutive TRs preceeding a feedback period exceeded this threshold, the trial was considered successful and a new trial began.

### Procedure: Visual display

We used a square-wave grating, 50% contrast, 0.5 cycles/degree, visible from 4–20° of visual angle. A black fixation circle was centered with a diameter of 0.5°. The green feedback circle was linearly mapped from 0–100% classifier output to 0.6–3.5°. A grey circle was displayed at 85% (3.065°) to indicate the success threshold. On training trials, the target was shown to participants so that they could learn how the feedback signal behaved. On these trials, a target orientation grating was shown (square-wave, 50% contrast, 0.5 cycles/degree, 4.5°), appearing underneath the feedback circle but on top of the stimulus grating, slightly covering it.

### Procedure: Continuous feedback

Continuous target search timings are summarized in Fig 3B. Each continuous target search began with the grating oriented horizontally (0°). Participants rotated the grating image using either the left (counter-clockwise) or right (clockwise) arrow keys (Fig 3A). While either key was depressed, the grating rotated at a constant rate of 45°/second. The grating did not rotate if neither or both keys were pressed. The V1 model was updated every two seconds based on the mean grating orientation over the previous two seconds. At each update period, the feedback circle expanded or contracted over 500ms from the previous value to the updated value to ensure a smooth visual display. If the average feedback signal exceeded the success threshold circle for three consecutive TRs, the target was considered reached and participants saw their grating orientation in grey overlaid with the true target orientation in green (Fig 3B).

Continuous target searches were organized into four blocks, with each block using one of the four candidate physiological filters. The first block was always the impulse condition, because this was the easiest condition and served as familiarization for participants. The next three blocks were the three other physiological filters: HRF, blur, and delay. Ordering of these three blocks was counterbalanced across participants. Each block began with three visible training targets at 45°, 90°, and 135°, so that participants could familiarize themselves with the feedback signal dynamics. This was followed by 14 hidden targets, randomly located at 0°, 22.5°, …, 157.5°, with two targets per orientation. A self-paced rest period was provided between each block, with an indication that the feedback signal behavior was about to change. Each block lasted about 10 to 20 minutes, depending on participant ability and the difficulty of each physiological filter.

### Procedure: Intermittent feedback

Intermittent trial timings are summarized in Table 1, with non-accelerated timings illustrated in Fig 3C. Each intermittent target began with the grating oriented horizontally (0°), and participants had three seconds to select an initial orientation. During this orientation selection period, the grating was greyed out (25% contrast) and rotated at a constant rate of 60°/second according to participants’ button presses. Rotation speed was increased relative to continuous feedback to allow for a full 180° of rotation during the 3 second adjustment period. Next, the stimulus was displayed at full contrast (50%) for a stimulus period whose duration depended on the feedback acceleration factor. Following a variable-length delay period (depending on feedback acceleration), feedback associated with the stimulus was presented for two seconds. The feedback signal was calculated by stimulating the V1 model with the selected orientation for 3 TRs (6 seconds), then averaging the feedback signal recorded in the following 3 TRs to account for the physiological delay. If the feedback signal exceeded the success threshold, participants saw their selected grating orientation in grey overlaid with the true target orientation in green (Fig 3C). If the feedback score did not reach the success threshold, a new trial with the same target began after a brief (depending on feedback acceleration) wait period. Starting from the second trial for a given target, the feedback display period began with a smooth 500ms update from the previous trial’s feedback value. Intermittent trials were organized into seven blocks with nine targets each. Targets were randomly located at 0°, 22.5°, …, 157.5°, with nine targets per orientation. The first three targets of the first block had the target visible to familizarize participants. All remaining targets were hidden. A self-paced rest period was provided between each block.

**Table 1:** Intermittent trial timings.

Acceleration	Select	Stimulus	Delay	Feedback	Wait	Total
Real-time	3 sec	6 sec	6 sec	2 sec	3 sec	20 sec
2x	3 sec	3 sec	3 sec	2 sec	2 sec	13 sec
6x	3 sec	1 sec	1 sec	2 sec	1 sec	8 sec

### Procedure: Accelerated intermittent feedback

We introduced and validated an accelerated intermittent feedback protocol to reduce experimental duration while preserving the trial-by-trial intermittent feedback signal characteristics. Accelerated intermittent trial timings are summarized in Table 1. Our validation used only the HRF physiological response filter. Targets were randomly and evenly assigned one of three trial timings: real-time, 2x accelerated, and 6x accelerated. The real-time timing corresponded to the required timing if a real physiological signal were measured: 6 seconds of stimulus, 6 seconds of waiting for the physiological signal to catch up, and 6 seconds between the end of feedback and the beginning of the next stimulus to allow the physiological signal to return to baseline. The 2x and 6x acceleration factor trials had the stimulus and subsequent wait period sped up accordingly. While these resulted in shorter trials (13s and 8s respectively), the trial-by-trial feedback signal was still simulated according to the real-time protocol, meaning that there was no difference in the trial-by-trial information observed by participants. This allowed us to investigate whether performance was affected by delays between stimulus and feedback. Participants (n = 10) were not informed about the different acceleration factors, which changed on a target-by-target basis but were constant for all trials on a given target. We constructed a linear mixed effects model of trials to target using feedback acceleration as a fixed effect and participant as a random effect. There was no significant effect of acceleration factor (F = 0.239, p = 0.788). Thus, all remaining intermittent feedback participants (n = 14) were exposed to the 6x acceleration factor.

### Procedure: Intermittent feedback with different physiological responses

In this intermittent feedback protocol, the HRF, delay, and blur physiological filters were used. The impulse filter was not used because this signal is not captured by our intermittent feedback calculation and participants cannot achieve an above-chance classifier output to reach the target. Targets were randomly and evenly assigned one of the three physiological filters. Unlike the continuous feedback condition, participants were not informed about the different filters because the feedback timing was identical between conditions, although signal quality did vary based on the physiological filter used.

Because we found that accelerating feedback did not significantly affect performance at the task, all trials in the intermittent feedback condition used the 6x acceleration factor, allowing us to record more trials in less time. When calculating the time that participants took to reach targets, we always used the non-accelerated time (20s per trial), because this is representative of how long the experiment would actually take if it were conducted inside the MRI scanner.

### Statistical analysis: Cognitive neurofeedback performance

Our outcome measure for performance on each trial was the amount of time needed to find the hidden target. For continuous and intermittent feedback, we constructed separate linear mixed effects models to predict time-to-target using physiological response as a fixed effect and participant as a random effect. Tukey’s post-hoc test (*α* <0.05) was used to determine the differences between each condition. For intermittent feedback, we could not directly measure time-to-target as in the continuous condition because of the feedback acceleration factor. Instead, we calculated this performance metric by counting the number of trials needed to reach the target and multiplying this by 20 seconds (the ‘real-time’ cost of each trial), and subtracting 5 seconds of time cost because there is no wait period at the beginning of the first trial (3 seconds) and success occurs as soon as the calculated feedback score exceeds the threshold (2 seconds from the final feedback period). To compare continuous and intermittent feedback, we performed two sample t-tests for each physiological response filter, comparing between the continuous and intermittent feedback groups.

### Model parameters: Automatic learning

We constructed a new 3-way classifier to emulate Shibata et al. (2011) [25], which used three target orientations: 10°, 70°, and 130° (Fig 2C). We used only 3 orientations because our learning model was unable to learn the non-convex objective function associated with the 8-way classifier used in our cognitive experiment. For training our pattern decoder, we simulated 210 training examples (70 of each orientation) with an SNR of 2, using the same V1 model described earlier (tuned to 8 orientations). For our real-time simulation, we also used an SNR of 2 (*σ*_*n*_ = 0.5). Unlike our cognitive simulation, where spontaneous activity corrupted the feedback signal and was treated as noise, here we leveraged this ‘noise’ whenever it spontaneously matched the desired pattern of activity. Therefore, we did not have to increase the SNR as we did in the cognitive experiment in order to make the signal learnable. Also unlike our cognitive experiment, we did not apply a tuning curve to the classifier outputs because we wanted to match the feedback signal presented by Shibata et al. (2011) [25].

### Procedure: Automatic learning simulation

We simulated five hours of neurofeedback training per simulated participant with a learning rate of 1% per 20 seconds, roughly matching the time scale of learning found by Shibata et al. (2011) [25]. For intermittent feedback simulations, this corresponded to a rate of 1% per trial; for continuous simulations, this corresponded to a rate of 0.1% per 2 second TR.

All four physiological filters from the cognitive experiment (Fig 1E) were used in the automatic simulation. We also had five possibilities for internal models (Fig 5A). For continuous feedback trials (Fig 5B), we used the four physiological filters for our four possible internal models: each matched filter should capture the time series of neural activity that resulted in the continuous feedback signal. For example, if the true physiological filter is an HRF, an internal model of HRF would apply the feedback to a memory trace of the preceding neural activity that gave rise to the feedback signal (Fig 5D). For intermittent feedback trials (Fig 5C), we used a single internal model generated by the cue-wait-feedback trial structure: activity during the 6-sec cue period is kept in memory and reinforced each trial based on the change in the delayed feedback signal. The cue structure also ensured that conditioned activity was only elicited during the cue period (Fig 5E). The intermittent feedback signal was calculated using an average of the recorded response during the wait period, similar to cognitive intermittent trials. Therefore, there were 20 total conditions simulated: 16 for continuous feedback with four physiological filters and four possible internal models, and 4 for intermittent feedback with only one internal model (cue). Because the simulated time series was non-deterministic due to spontaneous activity, we simulated each condition 1000 times to determine the distribution of responses at each time point over the course of learning.

### Statistical analysis: Automatic learning

The learning quality for each condition (combination of physiological filter and internal model) was evaluated based on the mean and the 50% confidence interval (CI) of the classifier output at each time point, combined across all simulated participants (1000 per condition). Over the course of 5 hours of simulated neurofeedback training, simulated participants were categorized into four categories: successful learning, trending learning, no learning, and anti-learning. Successful learning curves had a 50% CI ending well above chance. Trending learning curves increased over time, but at a much slower rate than than successful curves, and had a 50% CI that extended into chance at the end of training. Curves with no learning were flat. Anti-learning curves had a 50% CI that ended below chance. Because these classifications were arbitrary, we further ranked internal models for each physiological filter by the average final value of the classifier at the end of training. Pearson’s *r* was used to assess correlations between conditioned patterns, desired activity patterns, and classifier weight maps. Mean correlations with conditioned patterns were calculated by taking the correlation across 1000 examples and taking the mean of these correlations (e.g. not a single correlation with the mean conditioned pattern).
